# [Corrigendum] Cross regulation of signaling pathways in gastrointestinal stromal tumor

**DOI:** 10.3892/ol.2023.14103

**Published:** 2023-10-16

**Authors:** Yijun Qi, Wendi Zhao, Zhengguang Wang, Qingsong Xie, Jing Cao, Xiangling Meng

Oncol Lett 16: 6770–6776, 2018; DOI: 10.3892/ol.2018.9494

Subsequently to the publication of this paper, the authors contacted the Editorial Office to explain that errors had been made in terms of the assembly of Fig. 1, Fig. 2, Fig. 3; essentially, in Figs. 1 and 2, the Gli-1 data were set out incorrectly in the various figure parts, and in all three figures, owing to errors made during the filing of the data, the incorrect α-tubulin data were shown.

The revised versions of Figs. 1, 2 and 3, containing the reorganized data for the Gli-1 blots and the correct data for the α-tubulin blots, are shown opposite and on the next page. The authors regret the errors that were made in the compilation of the original figures, and are grateful to the editor of *Oncology Letters* for allowing them the opportunity to publish a Corrigendum. Furthermore, they apologize to the readership for any inconvenience caused.

## Figures and Tables

**Figure 1.**
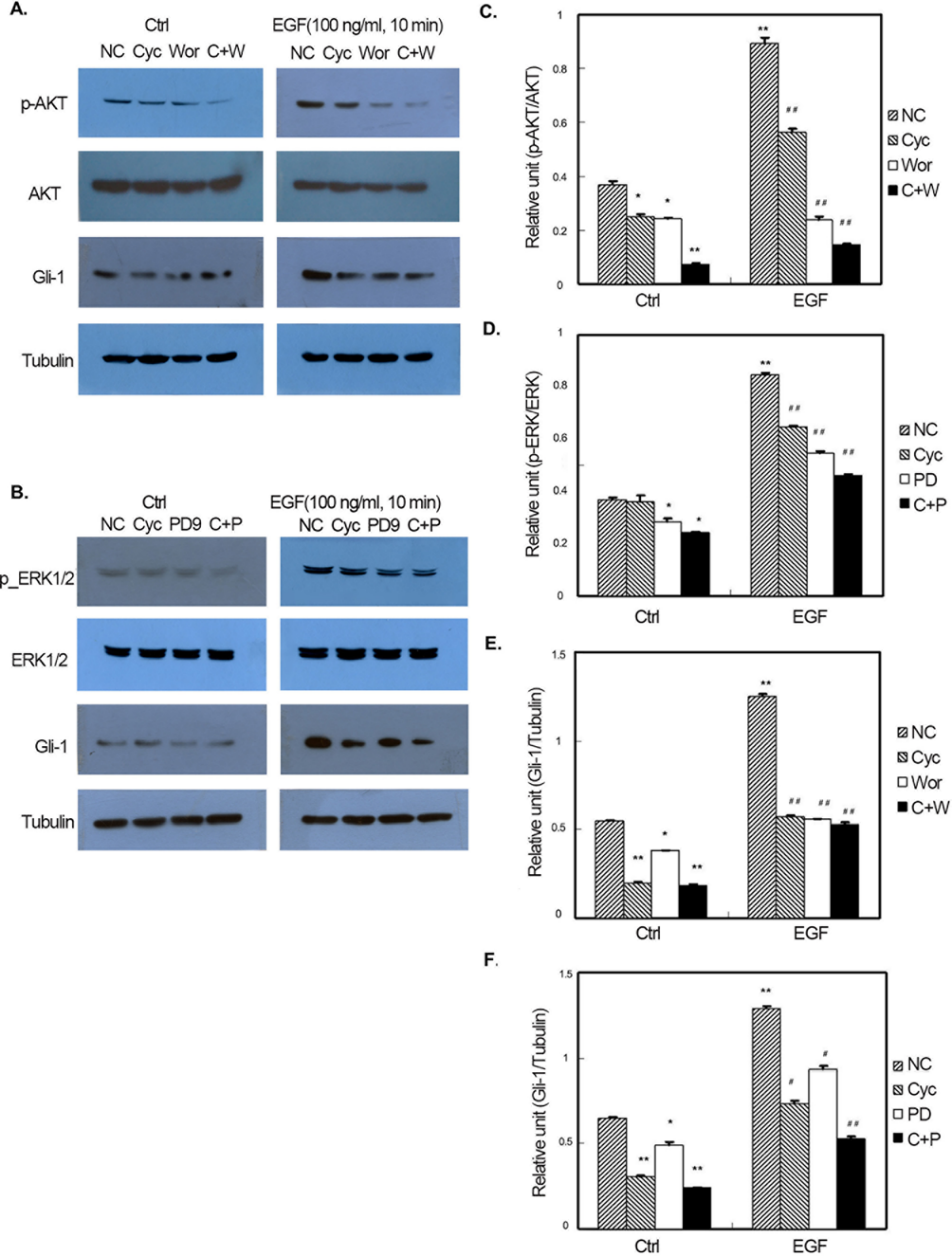
Effect of N-Shh on GIST-H1 cell signaling pathways. (A and B) Representative gels and quantification of (C) the effect of N-Shh on GIST-H1 cell AKT activation and the comparison of the effect from each treatment factor; (D) the effect of N-Shh on GIST-H1 cell ERK activation and the comparison of the effect from each treatment factor; (E) the effect of N-Shh on GIST-H1 cell Gli-1 expression and the comparison of the effect from each treatment factor; and (F) the effect of N-Shh on GIST-H1 cell Gli-1 expression and the comparison of the effect from each treatment factor (*P<0.05, **P<0.01 vs. ctrl-NC; ^#^P<0.05, ^##^P<0.05 vs. N-Shh-NC). N-Shh, recombinant Sonic hedgehog; GIST, gastrointestinal stromal tumor; EGF, endothelial growth factor; Gil-1, glioma-associated oncogene-1; NC, normal control; Cyc, cyclopamine; PD, PD98059; C+P, CPN-KAAD+PD98059; Wor, wortmannin; C+W, CPN-KAAD + wortmannin.

**Figure 2.**
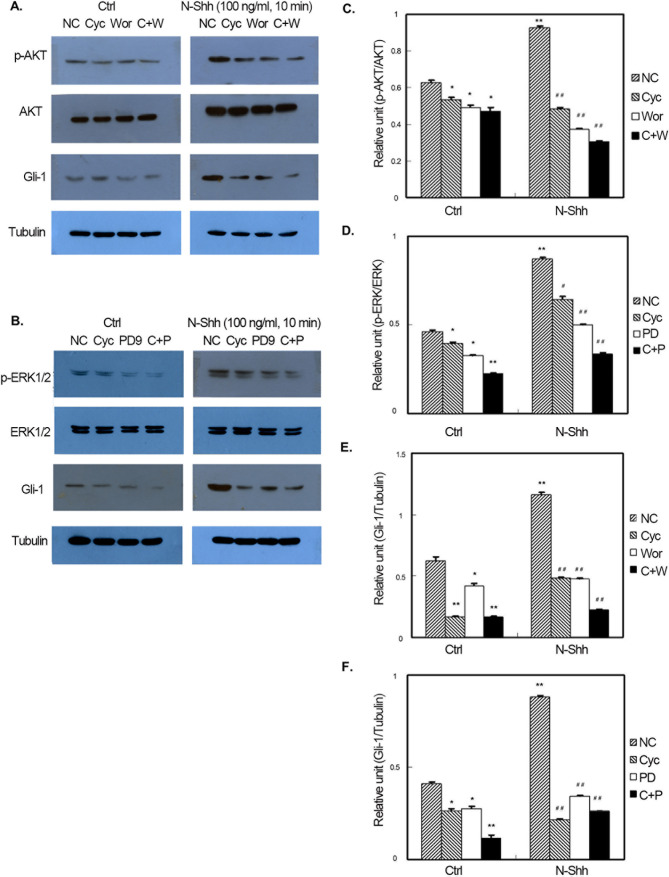
Effect of N-Shh on GIST-H1 cell signaling pathways. (A and B) Representative gels and quantification of (C) the effect of N-Shh on GIST-H1 cell AKT activation and the comparison of the effect from each treatment factor; (D) the effect of N-Shh on GIST-H1 cell ERK activation and the comparison of the effect from each treatment factor; (E) the effect of N-Shh on GIST-H1 cell Gli-1 expression and the comparison of the effect from each treatment factor; and (F) the effect of N-Shh on GIST-H1 cell Gli-1 expression and the comparison of the effect from each treatment factor (*P<0.05, **P<0.01 vs. ctrl-NC; ^#^P<0.05, ^##^P<0.05 vs. N-Shh-NC). N-Shh, recombinant Sonic hedgehog; GIST, gastrointestinal stromal tumor; EGF, endothelial growth factor; NC, normal control, Cyc, cyclopamine, PD, PD98059, C+P, CPN-KAAD+PD98059, Wor, wortmannin, C+W, CPN- KAAD+wortmannin.

**Figure 3.**
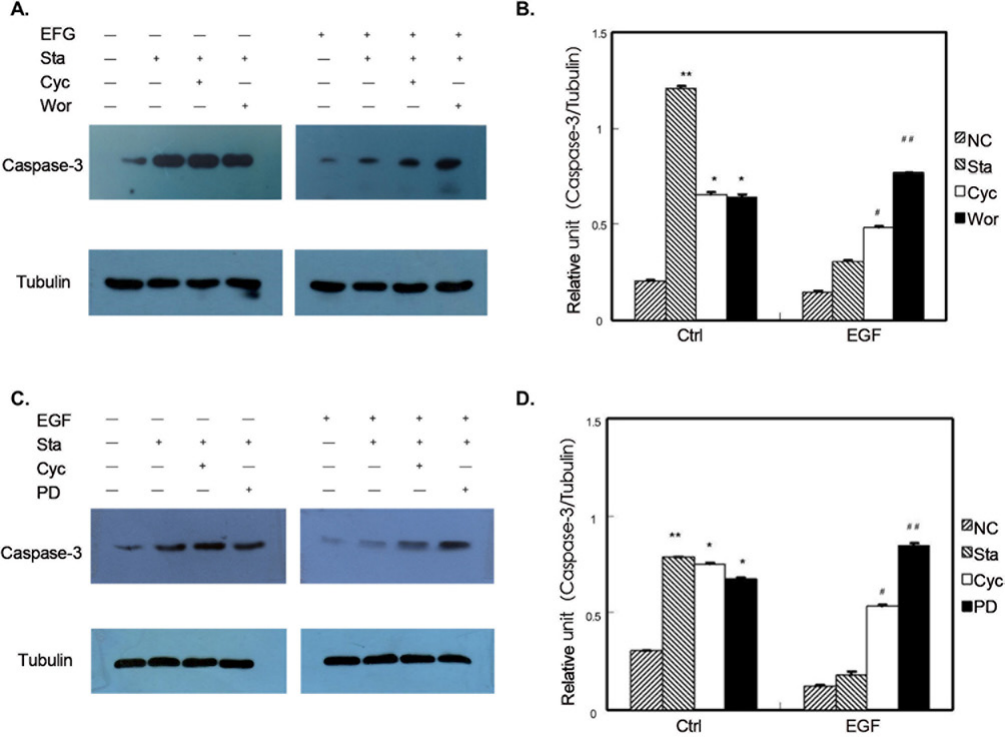
Effect of inhibiting Shh, PI3K and (or) MAPK signaling pathway on the expression of caspase-3 in GIST cells. (A) The influence on the expression of caspase-3 by inhibiting Shh and (or) PI3K signaling pathway in GIST cells. (B) The caspase-3/Tubulin relative intensity ratios of each treatment factor resulted from EGF-mediated cell apoptosis actions by inhibiting Shh and (or) PI3K signaling pathway. (C) The influence on the expression of caspase-3 by inhibiting Shh and (or) MAPK signaling pathway in GIST cells. (D) The caspase-3/Tubulin relative intensity ratios of each treatment factor resulted from EGF-mediated cell apoptosis actions by inhibiting Shh and (or) MAPK signaling pathway (*P<0.05, **P<0.05 vs. ctrl-NC; ^#^P<0.05, ^##^P<0.05 vs. EGF-NC). N-Shh, recombinant Sonic hedgehog; GIST, gastrointestinal stromal tumor; EGF, endothelial growth factor; NC, normal control, Sta, starvation, Cyc, cyclopamine, PD, PD98059, Wor-wortmannin.

